# Compressive Properties and Hydraulic Permeability of Human Meniscus: Relationships With Tissue Structure and Composition

**DOI:** 10.3389/fbioe.2020.622552

**Published:** 2021-02-10

**Authors:** Andy Morejon, Christopher D. Norberg, Massimiliano De Rosa, Thomas M. Best, Alicia R. Jackson, Francesco Travascio

**Affiliations:** ^1^Department of Mechanical and Aerospace Engineering, University of Miami, Coral Gables, FL, United States; ^2^Department of Biomedical Engineering, University of Miami, Coral Gables, FL, United States; ^3^Department of Orthopaedic Surgery, University of Miami, Coral Gables, FL, United States; ^4^UHealth Sports Medicine Institute, Coral Gables, FL, United States; ^5^Max Biedermann Institute for Biomechanics at Mount Sinai Medical Center, Miami Beach, FL, United States

**Keywords:** fibrocartilage, aggregate modulus, poisson modulus, confined compression, stress- relaxation, finite element modeling, glycosaminoglycans

## Abstract

The meniscus is crucial in maintaining knee function and protecting the joint from secondary pathologies, including osteoarthritis. The meniscus has been shown to absorb up to 75% of the total load on the knee joint. Mechanical behavior of meniscal tissue in compression can be predicted by quantifying the mechanical parameters including; aggregate modulus (*H*) and Poisson modulus (ν), and the fluid transport parameter: hydraulic permeability (*K*). These parameters are crucial to develop a computational model of the tissue and for the design and development of tissue engineered scaffolds mimicking the native tissue. Hence, the objective of this study was to characterize the mechanical and fluid transport properties of human meniscus and relate them to the tissue composition. Specimens were prepared from the axial and the circumferential anatomical planes of the tissue. Stress relaxation tests yielded the *H*, while finite element modeling was used to curve fit for ν and *K*. Correlations of moduli with water and glycosaminoglycans (GAGs) content were investigated. On average *H* was found to be 0.11 ± 0.078 MPa, ν was 0.32 ± 0.057, and *K* was 2.9 ± 2.27 × 10^−15^ m^4^N^−1^s^−1^. The parameters *H*, ν, and *K* were not found to be statistically different across compression orientation or compression level. Water content of the tissue was 77 ± 3.3% while GAG content was 8.79 ± 1.1%. Interestingly, a weak negative correlation was found between *H* and water content (R^2^ ~ 34%) and a positive correlation between *K* and GAG content (R^2^ ~ 53%). In conclusion, while no significant differences in transport and compressive properties can be found across sample orientation and compression levels, data trends suggest potential relationships between magnitudes of H and K, and GAG content.

## Introduction

Osteoarthritis (OA) is a degenerative joint disorder that has high socioeconomic impact, affecting 1 in 2 adults and resulting in more than $100 billion annually in the United States alone (Murphy and Helmick, 2012). The knee is highly susceptible to OA degenerative changes, and, in fact, is the most commonly affected joint. The fibrocartilaginous menisci are semilunar shaped tissues that are sandwiched between the femoral condyle and the tibial plateau in the knee. The primary function of the meniscus is to provide load distribution and transmission, while also maintaining joint congruency and lubrication. Importantly, it has been shown the menisci bear 45–75% of the total knee joint load, which can range from 2.7 to 4.9 times the body weight (Paul, 1976; Shrive et al., 1978). Therefore, damage or degeneration of the meniscus is detrimental to the underlying articular cartilage, and can lead to the progression of OA.

The ability of the menisci to perform its mechanical function is tied to its particular structure and composition. The extracellular matrix (ECM) of the tissue is composed primarily of collagen and proteoglycans, and ~75% of the tissue wet weight is water (Athanasiou and Sanchez-Adams, 2009). Collagen fibers are arranged in large bundles that are circumferentially aligned and held together by radial tie fibers, thereby providing high tensile stiffness. Areas of high proteoglycan content are able to withstand large compressive forces as well, since glycosaminoglycans (GAGs) attached to proteoglycans are responsible for imbibing the tissue with water, thereby increasing compressive stiffness (Athanasiou and Sanchez-Adams, 2009). Thus, the meniscus acts as a viscoelastic material that withstands forces in tension, compression, and shear in order to fulfill its mechanical function with the joint. Knowledge of mechanical properties of the native meniscus tissue can be used for designing tissue engineered replacements as well as for the development of computational models of the tissue, which can lead to new treatment modalities aimed at curbing OA in the joint.

Numerous investigators have studied the biomechanical behavior of human and animal menisci in tension, compression, and shear, see review (Athanasiou and Sanchez-Adams, 2009). Many of these studies have shown that mechanical properties of the tissue are anisotropic and inhomogeneous. The compressive properties of a tissue can be characterized by the aggregate modulus (*H*), Poisson ratio (ν), and the hydraulic permeability (*K*). Hydraulic permeability is also a measure of transport behavior in the tissue as it quantifies fluid movement through the ECM. Several studies have investigated these properties in human and animal menisci (Joshi et al., 1995; LeRoux and Setton, 2002; Sweigart et al., 2004; Sweigart and Athanasiou, 2005; Chia and Hull, 2008; Bursac et al., 2009; Seitz et al., 2013; Abdelgaied et al., 2015; Danso et al., 2018; Kleinhans and Jackson, 2018; Warnecke et al., 2020). However, there is limited information regarding their relation with tissue composition and structure. A better understanding of such relationships may provide key insights into meniscus physiology and pathogenesis, and can also be deployed in developing new tissue regeneration strategies.

In this study, we aimed to characterize the compressive properties of human meniscus tissues. We performed stress relaxation experiments of tissue plugs in confined compression that, combined with finite element modeling, yielded values for the aggregate modulus, Poisson's ratio, and hydraulic permeability. In order to determine the anisotropic nature of the tissue's biomechanics in compression, we used specimens having either circumferential (i.e., parallel to the collagen fiber bundles) or axial (i.e., orthogonal to fibers) orientations. In addition, we also investigated the strain-dependent behavior by varying the magnitude of compressive strain from 5 to 20%. Finally, we measured the composition of the tissue (e.g., water, glycosaminoglycan contents) in order to determine if a relationship exists between mechanical properties and tissue composition. This study provides important quantitative information and structure-function relations that can be employed for computational modeling or tissue engineering applications to develop new treatments for meniscus degeneration and related OA.

## Methods

### Sample Preparation

**Figure 1:**
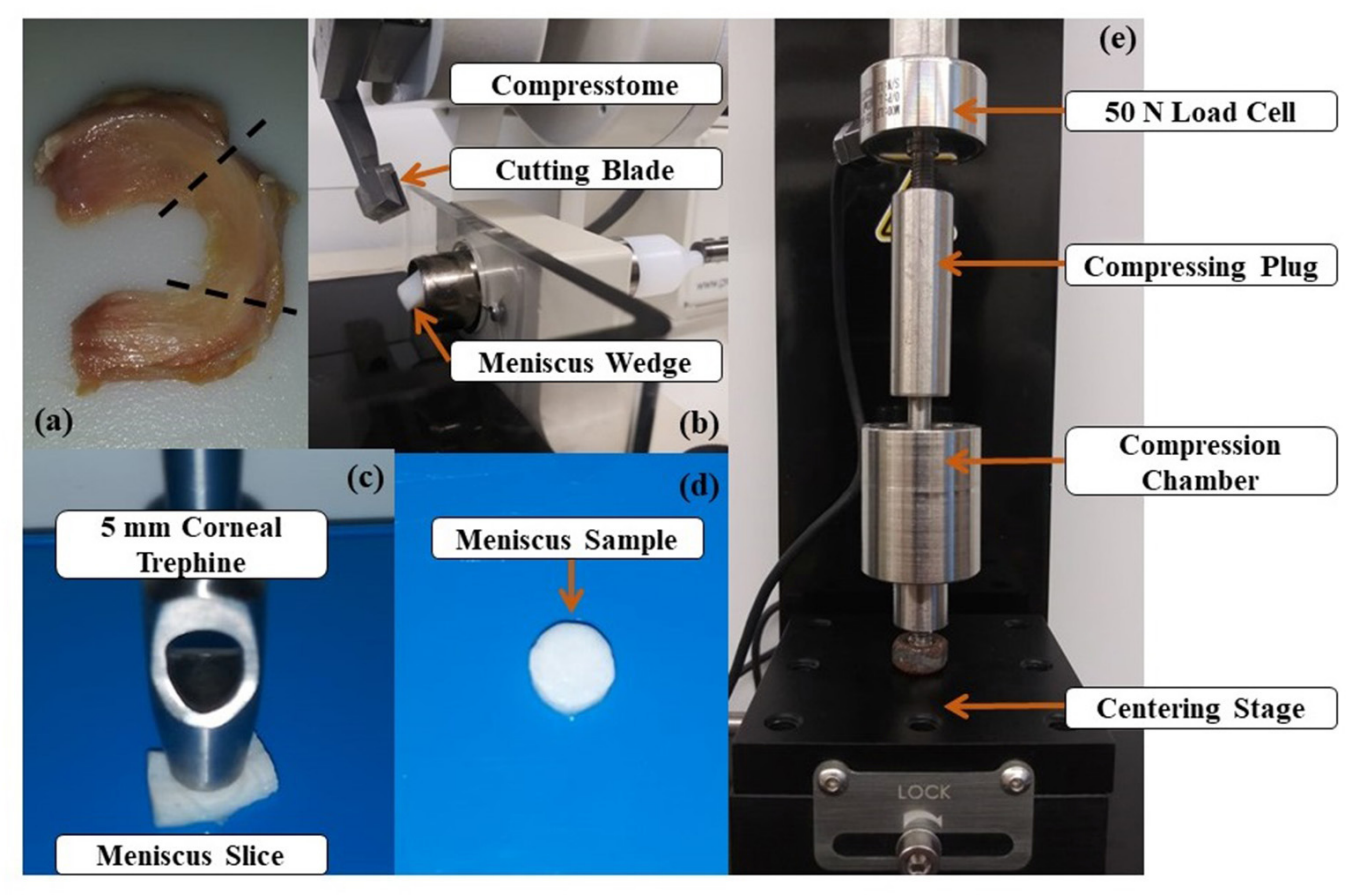
Specimen preparation and experimental apparatus: **(a)** Human meniscus specimen. The tissue region where samples are taken from is included between the dashed lines; **(b)** Meniscus wedges are sliced via Compresstome^®^ **(c)** A 5 mm trephine cut the meniscal slice in a cylindrical specimen; **(d)** Meniscus specimen used in the experiments; **(e)** Uniaxial testing apparatus used for confined compression testing.

Three lateral and 5 medial human menisci were obtained frozen from five cadavers aged 73.5 ± 6.4 y.o. The central regions of the menisci were sectioned into wedges before cut in 1.5 mm slices in axial and circumferential directions using a compresstome^®^ (VF-200-0Z, Precisionary, Natick, MA). A 5 mm diameter corneal trephine was used to cut at the center of the slices into 1.39 ± 0.32 mm tall cylinders, see Figure 1. A total of three samples were prepared per meniscus: two for stress-relaxation experiments (axial and circumferential) and one for water and GAG content measurements taken adjacent to the axial sample. Once prepared, samples designated for mechanical tests were stored at −4°C submerged in protease inhibited (Complete Tablets, Roche, Basel, SWI) 1X phosphate buffered saline (PBS) solution until testing. Samples designated for measurements of tissue composition were immediately processed as detailed below.

### Mechanical Testing

Experiments were conducted to measure stress-relaxation during confined compression. The cylindrical samples were thawed at room temperature and placed inside the 5 mm diameter compression chamber filled with PBS. A uniaxial testing apparatus (Univert, Cell Scale, Waterloo, ON) with a 50 N load cell was used to measure the normal force during testing, see Figure 1. For each sample, a total of three consecutive stress-relaxation tests were carried out at 5, 10, and 20% strains. The samples were compressed by a porous plug that allowed for fluid exudation during compression. The compressive strains were calculated based on the height of the sample calculated after applying a preload of 0.1 N. The preload was used to ensure proper contact at the interface between tissue and porous plug. Each compression sequence consisted of a 1 s ramp followed by 3,000 s hold period. Preliminary data and similar studies have shown that 3,000 s is sufficient for load equilibrium to occur in meniscal tissue under compression (Williamson et al., 2001; Seitz et al., 2013). All data were recorded at 5 Hz sampling frequency using Labjoy (Version 10.78, Cellscale.com). The aggregate modulus (*H*) was calculated via in-house developed MATLAB^®^ v2019a (Mathworks, Inc., Natick, MA) script which divided the value of the normal force at equilibrium by the applied strain applied and the cross-sectional area of the sample (Chia and Hull, 2008; Bursac et al., 2009; Seitz et al., 2013). Both Poisson's ratio (ν) and hydraulic permeability (*K*) were calculated by curve-fitting the stress-relaxation experimental data with the numerical solution of a finite element model of a meniscal sample undergoing confined compression. Curve-fitting was carried out using a Levenberg-Marquardt algorithm implemented in the Parameter Optimization Module (Version 2.0, febio.org) included in FEBio Studio (Version 1.0, febio.org).

### Finite Element Model

**Figure 2:**
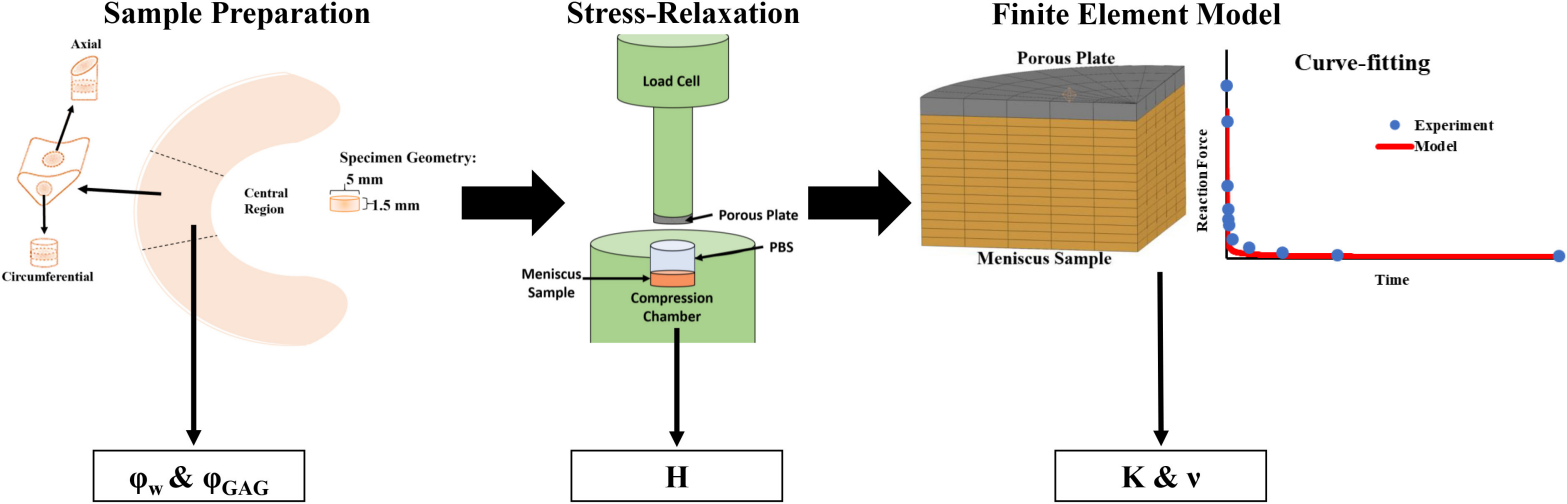
Summary of experimental procedures. The samples were prepared into 5 mm diameter disks which were used for mechanical and biochemical testing. Confined stress-relaxation experiments were conducted on axial and circumferential samples (*n* = 8) which yielded *H* from measurement of the residual reaction force on the compressing porous plug. Curve-fitting of stress-relaxation tests with FEM model yielded values of ν and *K*.

A computational model for confined compression of meniscal samples was implemented in FEBio Studio (Version 1.0, febio.org) (Maas et al., 2012). The meniscal tissue was modeled as a poroelastic continuum according to established theoretical frameworks (Bowen, 1980; Mow et al., 1980). Specifically, as previously reported, the meniscus solid phase was described as characterized by an isotropic neo-Hookean constitutive equation (Seitz et al., 2013). The fluid phase embedding the solid matrix was assumed inviscid and incompressible. Fluid percolation through the porous solid phase was governed by Darcy's law with constant, isotropic *K*. The compressing porous plug was modeled as a rigid body. Shape and dimension of the modeled tissue were the same as those of the specimens tested. Aimed at simulating a confined compression, the bottom of the sample, as well as its lateral surface were considered impermeable and fixed, so as to prevent fluid exudation and solid displacement. The fluid could only be exchanged through the superior surface, which was displaced by the porous plug with the same compression sequence used in the experiments. Based on a preliminary mesh convergence study, a total of 600 8-node hexahedral elements were used to represent the meniscal sample. Model output was the time-dependent normal force on the compressing porous plug. A schematic of the computational model is reported in Figure 2.

### Measurement of Tissue Composition

Water and GAG content of menisci specimens were measured. An analytical balance (Model ML104, Mettler Toledo, Columbus, OH) was used to record the sample weight in air (*W*_*wet*_), in PBS solution (*W*_*s*_), and immediately after 24-h lyophilization (*W*_*dry*_). The water volume fraction, φ_w_ was calculated based on the following equation (Travascio et al., 2020):

(1) $$\varphi_{w}=\frac{(W_{wet}-W_{dry})\rho_{s}}{(W_{wet}-W_{s)\;}\rho_{w}}\%.$$

where ρ_*s*_ and ρ_*w*_ are the densities of PBS and water, respectively. Subsequently, the GAG content was measured using a 1,9-dimethylmethylene blue (DMMB) (Polysciences Inc., Warrington, PA) binding assay (Burton-Wurster et al., 2003). Following lyophilization, tissues were digested using papain solution (250 μg/ml). Once digested, tissues were mixed with DMMB and the absorbance was measured using a multi-mode microplate reader (Molecular Devices SpectraMax M2 Series, Sunnyvale, CA) at 525 nm wavelength. The GAG content (φ_GAG_) was defined as:

(2) $$\varphi_{GAG}=\frac{W_{GAG}}{W_{dry}}\%,$$

### Statistical Analysis

All data were reported as mean ± standard deviation. Data normality was investigated via Anderson-Darling tests (α = 0.05). One-way ANOVA tests (α = 0.05) were conducted to determine whether the magnitude of compression (5, 10, or 20%) had significant effects on *H, K* or ν. In addition, 2-sample *t*-tests were performed comparing the values of *H*, ν, and *K* measured in the axial specimens to those observed in the circumferential ones. Also, simple linear regression analyses were conducted to individuate possible empirical relations among the parameters *H*, ν, and *K* and water or GAG content. For all analyses conducted, Grubbs tests (α = 0.05) were conducted to identify any outliers.

## Results

**Figure 3:**
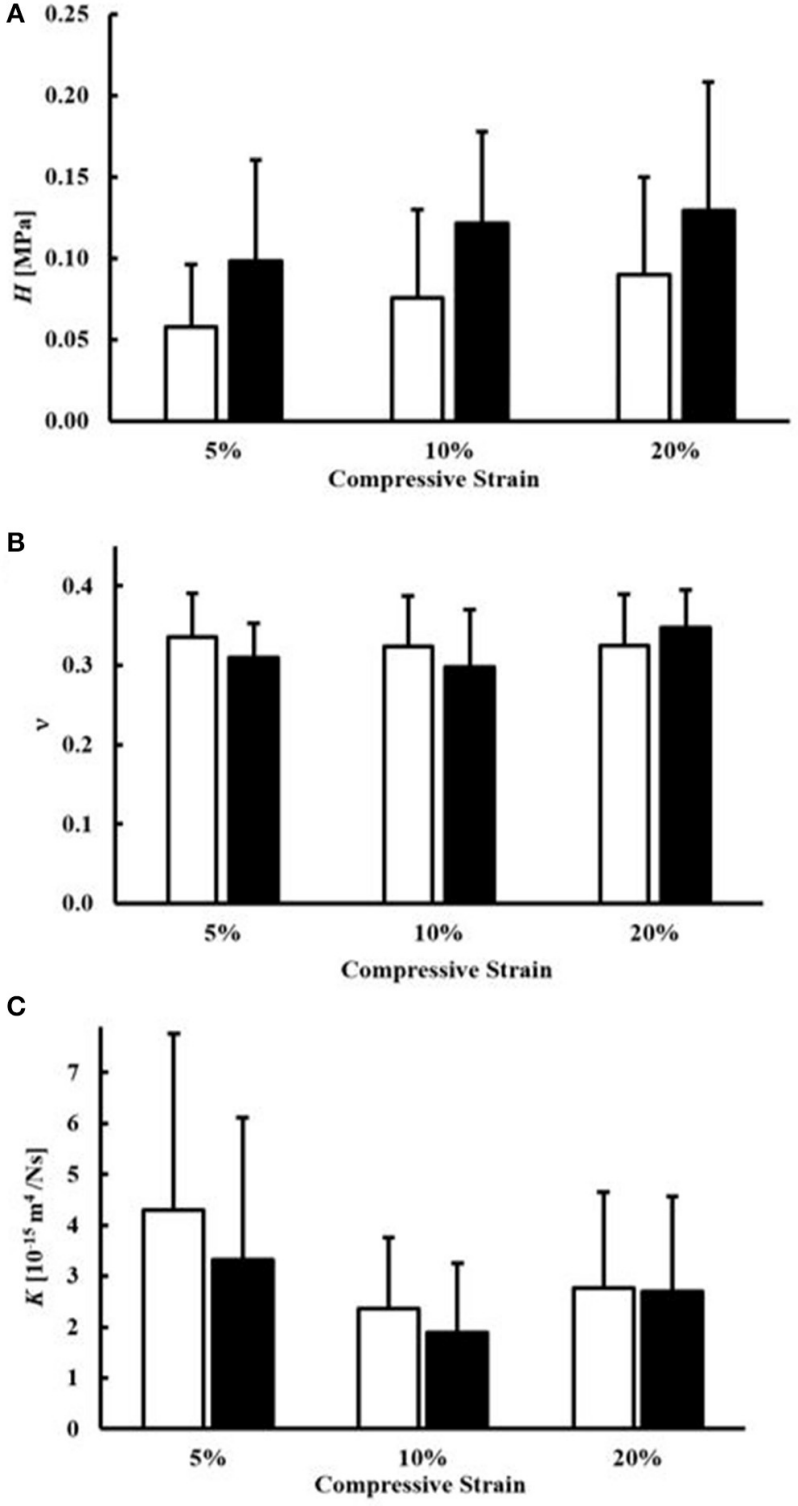
Mechanical and transport parameters of human meniscal tissue. Effects of specimen orientation and level of compression are illustrated for: **(A)** the modulus *H*, **(B)** the Poisson modulus ν and **(C)** the permeability *K*. For all the data presented, white bars refer to axial samples, and black bars refer to circumferential samples.

Measurement of *H*, ν, and *K* for all magnitudes of compression and sample orientation are reported in Figure 3. The magnitude of *H* did not significantly change with the magnitude of compression (*p* = 0.93). On average, circumferential samples were stiffer (*H* = 0.13 ± 0.078 MPa) than axial ones (*H* = 0.09 ± 0.073 MPa), although the differences were not statistically significant (*p* = 0.24) Also, the Poisson modulus (ν = 0.32 ± 0.057) did not significantly change with each magnitude of compression (*p* = 0.43) or tissue orientation (*p* = 0.66). Finally, no statistical differences were found in the values of *K* among difference magnitudes of compression (*p* = 0.078). For all the cases investigated, axial samples were characterized by larger values of *K* (3.1 ± 2.4 × 10^−15^ m^4^N^−1^s^−1^) when compared to circumferential ones (K = 2.6 ± 2.1 × 10^−15^ m^4^N^−1^s^−1^). However, the differences were not statistically significant (*p* = 0.506).

**Figure 4:**
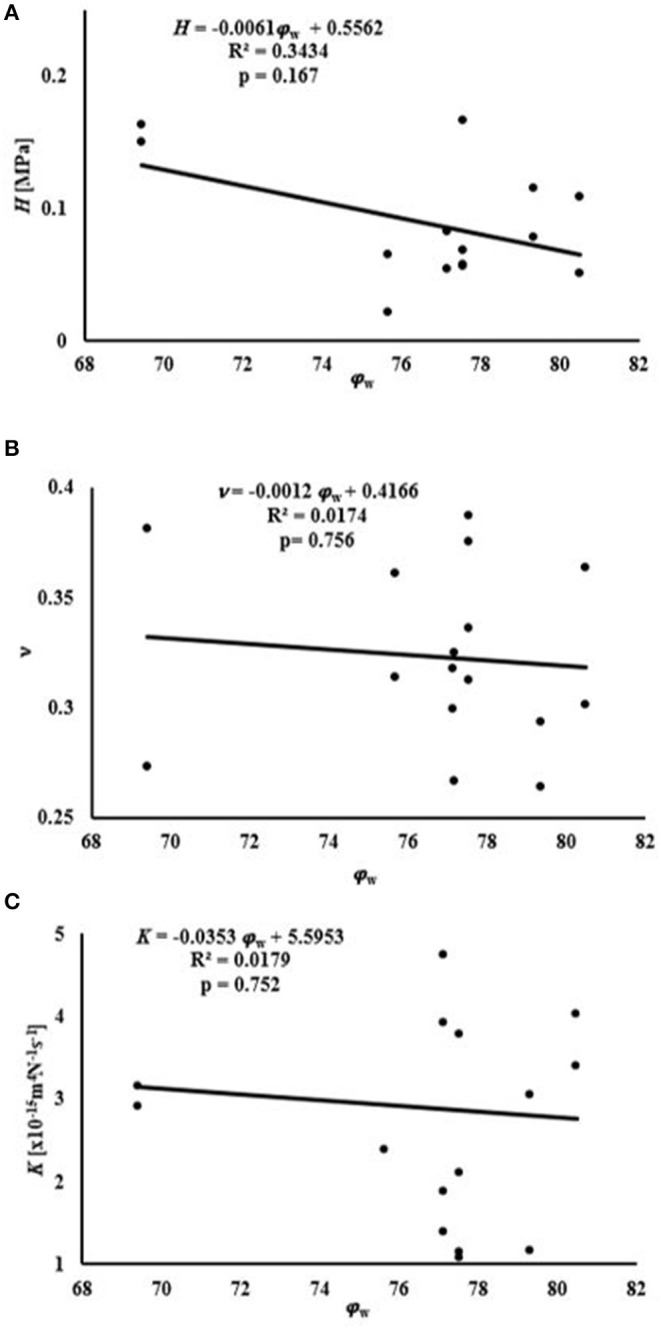
Relationships between mechanical and transport parameters and φ_w_: **(A)** relationship with *H*; **(B)** relationship with ν; **(C)** relationship with *K*.

**Figure 5:**
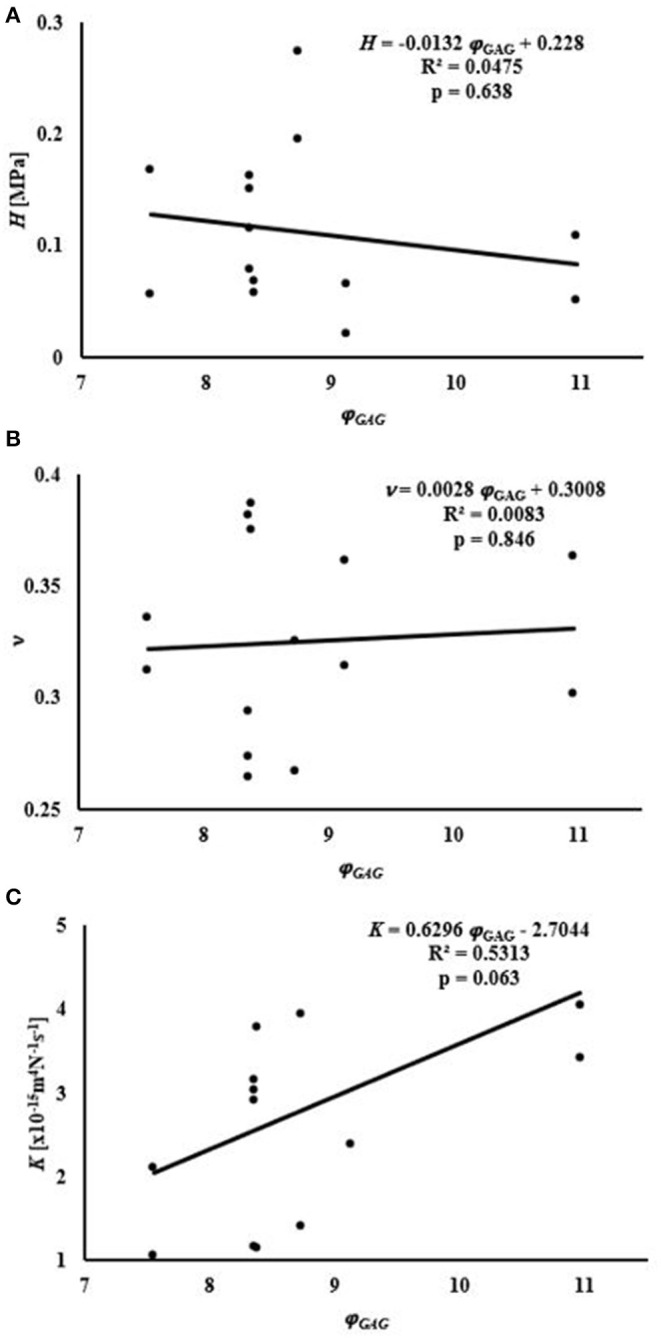
Relationships between mechanical and transport parameters and φ_GAG_: **(A)** relationship with *H*; **(B)** relationship with ν; **(C)** relationship with *K*.

Average water content of the samples was 76.8 ± 3.3% of the total weight, while that of GAGs was 8.79 ± 1.1% of the tissue dry mass, see Table 1. A summary of the empirical relations among the mechanical and transport parameters and tissue composition is reported in Figures 4, 5. The values of *H* decreased as water or GAG content increased. However, statistically significant relations were only found when relating *H* to water content in the axial orientation (*p* = 0.02). No statistically significant relations were found between *K* and water or GAG content. However, a trend of increasing values of *K* with GAG content was observed (*p* = 0.063). No correlation between Poisson's modulus and tissue composition was found.

**Table 1:** Summary of tissue composition.

**φ_w_**	**φ_GAG_**
76.79 ± 3.32%	8.79 ± 1.07%

*Values of water and GAG content are calculated according to Equations (1) and (2)*.

## Discussion

Stress-relaxation experiments were conducted in confined compression to yield values of *H*, ν, and *K* at magnitudes of compression ranging from 5 to 20%. These levels of compression were chosen as they represent physiological levels of loading in the meniscus (Chia and Hull, 2008; Yang et al., 2010). The results of this study show that compressive strains in this range did not significantly affect the magnitude of *H* for all cases investigated (*p* = 0.93). This is in agreement with previous studies on both human (Chia and Hull, 2008; Seitz et al., 2013) and animal (Sweigart et al., 2004; Abdelgaied et al., 2015) menisci being compressed from 3 to 20%. This finding would suggest that, within the physiological range of deformation explored in this study, the meniscal tissue exhibits a linear elastic behavior. This is the first study comparing the mechanical compressive behavior of human meniscus along the parallel and orthogonal directions of collagen fibers in the tissue. It was found that the average values of *H* in the axial and circumferential directions, 0.086 and 0.126 MPa, respectively, were not statistically different (*p* = 0.24), see Figure 3. This may be due to the fact that collagen fibers would contribute to the mechanical stiffness of the tissue only in tension.

Similar to our findings for the aggregate modulus, the values of the Poisson modulus did not significantly change with either compressive strain (*p* = 0.43) or sample orientation (*p* = 0.66), see Figure 3. The average value of ν measured was 0.32, which is within the range of previously reported measurements on bovine meniscus by Danso et al. (2018) via confined and unconfined compression tests. Other studies have reported much lower values of Poisson modulus (Sweigart et al., 2004; Abdelgaied et al., 2015); however, those studies focused on the mechanical characterization of the superficial layers of the tissue rather than the bulk properties, as performed here. Further investigation of the Poisson ratio in different layers within the same meniscus tissue would provide more insight into the heterogeneity of this tissue property.

The values for hydraulic permeability determined here were similar to those found in the literature for human and animal tissues, ranging from 0.07 to 6.8 × 10^−15^ m^4^N^−1^s^−1^ (Joshi et al., 1995; LeRoux and Setton, 2002; Sweigart et al., 2004; Seitz et al., 2013; Danso et al., 2018; Kleinhans and Jackson, 2018; Warnecke et al., 2020). Our results also showed that hydraulic permeability did not significantly vary with the magnitude of the compressive strain (*p* = 0.078), similar to an earlier study in human tissues with strain levels up to 20% (Seitz et al., 2013). In contrast, other studies have shown that *K* significantly decreases with compressive strain (Danso et al., 2015; Kleinhans and Jackson, 2018). Measurements from the present study only suggest a trend of *K* reducing with strain level, see Figure 3C. The discrepancy may be the result of a difference in measurement technique: while the present study and that by Seitz et al. (2013) employed a confined compression experiment, other studies used direct permeation (Kleinhans and Jackson, 2018) and indentation testing (Danso et al., 2015). In the present study, the mean values of *K* were 3.1 × 10^−15^ m^4^N^−1^s^−1^ and 2.6 × 10^−15^ m^4^N^−1^s^−1^ for the axial and circumferential orientations, respectively, suggesting that hydraulic permeability is slightly higher in the axial direction. To our knowledge, only one other study investigated the anisotropic behavior of permeability in meniscus tissues (Kleinhans and Jackson, 2018); that report found the opposite trend, with *K* being significantly higher in the circumferential direction as compared to the axial direction. However, key differences in that study compared to the current study, including use of healthy porcine tissues and measurement via direct permeation testing, likely contributed to the differences. More investigation is warranted in order to better understand the behavior of fluid transport in human meniscus tissues.

Measurements of tissue composition allowed for investigating any potential relationships among mechanical and transport parameters, and water and GAG contents. In this study, we focused on water and GAG contents because these are the key components known to support compressive loads in the tissue. The volumetric water content was found to be 76.8 ± 3.3%, which is within the range of water content measured in previous studies (Proctor et al., 1989; Joshi et al., 1995). The sulfated GAG content amounted to 8.79 ± 1% of the tissue dry weight, also consistent with previous GAG measurements (Herwig et al., 1984). A negative relationship was found between the *H* measured and the water content of the tissue. However, this relationship was significant in the compression of axial samples (*p* = 0.02). A similar result was also found in studies with meniscal compression in the axial orientation (Joshi et al., 1995; Bursac et al., 2009; Seitz et al., 2013). Stress on the ECM of porous tissue decreases as fluid leaves. Therefore, menisci with higher water content tend to allow the release of greater volumes of water which reduces stress on the ECM (Han et al., 2018).

No correlation was found between water content and *K*. Previous studies have found that hydraulic permeability trends positively with water content in meniscal tissue (Joshi et al., 1995; Seitz et al., 2013; Kleinhans and Jackson, 2018). However, in the current study such relationship was not found. The divergence among these findings may be related to interspecies variations; for example, the study by Joshi et al. found the strongest correlation between water content and permeability for porcine tissues, which is the same tissue source used by Kleinhans and Jackson. Furthermore, differences in specimen harvest locations, which influence the structural organization of the tissue, may also contribute to these differences. Numerous empirical models of hydraulic permeability in porous hydrated media also suggest a positive correlation with tissue water content. Therefore, more investigation is warranted, with a larger sample size, in order to better characterize this relationship.

The present study also found no correlation between H and GAG content for human meniscus tissues. Bursac et al. (2009) found a positive correlation between meniscal stiffness and GAG content. However, the meniscal samples used in their study were characterized by a GAG content lower than that observed in our experiments. Also, a regional variation in the contribution of GAGs to meniscal mechanical properties has been reported: presence of GAGs increases the compressive modulus in the inner and middle region of the meniscus, but has no effect on the outer part of the tissue (Sanchez-Adams et al., 2011). A study investigating the mechanical properties of different regions of the tissue over a wider range of GAG contents may provide more insight into the contribution of this component to the behavior of the meniscus in compression. In addition, a positive trend (*p* = 0.063) was found between K and GAG content. In articular cartilage, GAGs provide the greatest resistance to flow (Reynaud and Quinn, 2006) but the opposite effect was recorded in the present study. It is also known that GAG content in articular cartilage is larger than that in meniscus. This might suggest a different role this component may have in the mechanical behavior of the meniscus as opposed to that assumed in cartilage. Further investigation into this relationship is necessary to confirm this result. No relationship was found relating the Poisson modulus of the tissue to its biochemical contents (see Figures 4, 5). To the best of our knowledge, the effect of water and GAG contents on ν of meniscal tissue has not been previously recorded.

This study presented some limitations. Meniscal samples were obtained from elderly donors (73.5 ± 6.4 y.o.) who represent only a part of the general adult population. Age-related changes in the composition of the knee meniscus have been reported (Tsujii et al., 2017). Accordingly, the mechanical properties and their relationship with tissue structure and composition determined in this study may vary from those one would measure in a younger age group. Data from lateral and medial menisci were pooled together, although they are different in shape and size (Makris et al., 2011), as well as in biochemical composition (Adams et al., 1983; Seitz et al., 2013). While significant regional variations in tissue stiffness have been reported for porcine meniscus (Sweigart and Athanasiou, 2005), several other studies on both animal and human tissue (Seitz et al., 2013; Kleinhans and Jackson, 2018) did not find significant differences in mechanical and transport properties between medial and lateral menisci. In addition, only the core of the sample was analyzed, while the external layer of tissue was neglected. The external layer of the meniscus has different composition and structural organization when compared to its core (Proctor et al., 1989). Such differences may significantly affect both mechanical and transport parameters, warranting a dedicated study. Due to experimental procedures, specimens for composition measurements were taken from adjacent regions of the tissue; it is thus possible that stronger correlations may be found if direct measurements for composition were taken from experimental samples, as in earlier studies (Kleinhans and Jackson, 2018). For the purposes of model simplification, the computational model of the meniscus compression used to curve fit the experimental data assumed *K* to be isotropic and constant. Previous studies measuring *K* via direct permeation showed a dependence of such parameter with the magnitude of the compressive strain applied (Kleinhans and Jackson, 2018). In this study, *K* was assumed to be constant, which is consistent with an earlier study indicating that, in confined compression tests, a poroelastic model with constant *K* may better interpret the experimental data as compared to a mathematical representation including strain dependence (Seitz et al., 2013).

## Conclusion

In conclusion, results of the present study add new knowledge on *H*, ν, and *K* and their relationship with tissue structural organization and composition. Specifically, while no significant differences in transport and compressive properties can be found across sample orientation and compression levels, data trends suggest potential relationships between magnitudes of H and K, and GAG content. Such information can be utilized to better understand meniscal mechanics, mechanobiology and physiology, to develop computational models to predict meniscus mechanical behavior *in vivo*, as well as to design and develop meniscal replacements.

## Data Availability Statement

The raw data supporting the conclusions of this article will be made available by the authors, without undue reservation.

## Author Contributions

Research design was conducted by AM, CN, MD, TB, FT, and AJ. Data acquisition and analysis was carried out by AM, CN, MD, and FT. Data interpretation involved AM, FT, TB, and AJ. All the authors were involved in drafting and revising the manuscript, as well as reading and approving its final version.

## Conflict of Interest

The authors declare that the research was conducted in the absence of any commercial or financial relationships that could be construed as a potential conflict of interest.

## References

[B1] Abdelgaied A. Stanley M. Galfe M. Berry H. Ingham E. Fisher J. (2015). Comparison of the biomechanical tensile and compressive properties of decellularised and natural porcine meniscus. J. Biomech. 48, 1389–1396. 10.1016/j.jbiomech.2015.02.044 25766391

[B2] Adams M. E. Billingham M. E. Muir H. (1983). The glycosaminoglycans in menisci in experimental and natural osteoarthritis. Arthritis Rheum. 26, 69–76. 10.1002/art.1780260111 6401994

[B3] Athanasiou K. A. Sanchez-Adams J. (2009). Engineering the knee meniscus. Synthesis Lectures Tissue Eng. 1, 1–97. 10.2200/S00186ED1V01Y200903TIS001

[B4] Bowen R. M. (1980). Incompressible porous media models by use of the theory of mixtures. Int. J. Eng. Sci. 18, 1129–1148. 10.1016/0020-7225(80)90114-717887904

[B5] Bursac P. Arnoczky S. York A. (2009). Dynamic compressive behavior of human meniscus correlates with its extra-cellular matrix composition. Biorheology 46, 227–237. 10.3233/BIR-2009-0537 19581729

[B6] Burton-Wurster N. Liu W. Matthews G. L. Lust G. Roughley P. J. Glant T. T.. (2003). TGF beta 1 and biglycan, decorin, and fibromodulin metabolism in canine cartilage. Osteoarthritis Cartilage 11, 167–176. 10.1053/S1063-4584(02)00349-7 12623288

[B7] Chia H. N. Hull M. L. (2008). Compressive moduli of the human medial meniscus in the axial and radial directions at equilibrium and at a physiological strain rate. J. Orthop. Res. 26, 951–956. 10.1002/jor.20573 18271010

[B8] Danso E. K. Julkunen P. Korhonen R. K. (2018). Poisson's ratio of bovine meniscus determined combining unconfined and confined compression. J. Biomech. 77, 233–237. 10.1016/j.jbiomech.2018.07.001 30055840

[B9] Danso E. K. Mäkelä J. T. A. Tanska P. Mononen M. E. Honkanen J. T. J. Jurvelin J. S.. (2015). Characterization of site-specific biomechanical properties of human meniscus—Importance of collagen and fluid on mechanical nonlinearities. J. Biomech. 48, 1499–1507. 10.1016/j.jbiomech.2015.01.048 25708321

[B10] Han G. Hess C. Eriten M. Henak C. R. (2018). Uncoupled poroelastic and intrinsic viscoelastic dissipation in cartilage. J. Mech. Behav. Biomed. Mater. 84, 28–34. 10.1016/j.jmbbm.2018.04.024 29729578

[B11] Herwig J. Egner E. Buddecke E. (1984). Chemical changes of human knee joint menisci in various stages of degeneration. Ann. Rheum. Dis. 43, 635–640. 10.1136/ard.43.4.635 6548109 PMC1001426

[B12] Joshi M. D. Suh J. K. Marui T. Woo S. L. Y. (1995). Interspecies variation of compressive biomechanical properties of the meniscus. J. Biomed. Mater. Res. 29, 823–828. 10.1002/jbm.820290706 7593020

[B13] Kleinhans K. L. Jackson A. R. (2018). Hydraulic permeability of meniscus fibrocartilage measured via direct permeation: effects of tissue anisotropy, water volume content, and compressive strain. J. Biomech. 72, 215–221. 10.1016/j.jbiomech.2018.03.011 29605083

[B14] LeRoux M. A. Setton L. A. (2002). Experimental and biphasic FEM determinations of the material properties and hydraulic permeability of the meniscus in tension. J. Biomech. Eng. 124, 315–321. 10.1115/1.1468868 12071267

[B15] Maas S. A. Ellis B. J. Ateshian G. A. Weiss J. A. (2012). FEBio: finite elements for biomechanics. J. Biomech. Eng. 134:011005. 10.1115/1.4005694 22482660 PMC3705975

[B16] Makris E. A. Hadidi P. Athanasiou K. A. (2011). The knee meniscus: structure–function, pathophysiology, current repair techniques, and prospects for regeneration. Biomaterials 32, 7411–7431. 10.1016/j.biomaterials.2011.06.037 21764438 PMC3161498

[B17] Mow V. C. Kuei S. C. Lai W. M. Armstrong C. G. (1980). Biphasic creep and stress relaxation of articular cartilage in compression: theory and experiments. J. Biomech. Eng. 102, 73–84. 10.1115/1.3138202 7382457

[B18] Murphy L. Helmick C. G. (2012). The impact of osteoarthritis in the United States: a population-health perspective. AJN Am. J. Nurs. 112, S13–S9. 10.1097/01.NAJ.0000412646.80054.21 22373741

[B19] Paul J. P. (1976). Force actions transmitted by joints in the human body. Proc. R. Soc. Lond. B Biol. Sci. 192, 163–172. 10.1098/rspb.1976.0004 3785

[B20] Proctor C. S. Schmidt M. B. Whipple R. R. Kelly M. A. Mow V. C. (1989). Material properties of the normal medial bovine meniscus. J. Orthop. Res. 7, 771–782. 10.1002/jor.1100070602 2677284

[B21] Reynaud B. Quinn T. M. (2006). Anisotropic hydraulic permeability in compressed articular cartilage. J. Biomech. 39, 131–137. 10.1016/j.jbiomech.2004.10.015 16271597

[B22] Sanchez-Adams J. Willard V. P. Athanasiou K. A. (2011). Regional variation in the mechanical role of knee meniscus glycosaminoglycans. J. Appl. Physiol. 111, 1590–1596. 10.1152/japplphysiol.00848.2011 21903884 PMC3233877

[B23] Seitz A. M. Galbusera F. Krais C. Ignatius A. Dürselen L. (2013). Stress-relaxation response of human menisci under confined compression conditions. J. Mech. Behav. Biomed. Mater. 26, 68–80. 10.1016/j.jmbbm.2013.05.027 23811278

[B24] Shrive N. G. O'connor J. J. Goodfellow J. W. (1978). Load-bearing in the knee joint. Clin. Orthop. Relat. Res. 131, 279–287. 10.1097/00003086-197803000-00046 657636

[B25] Sweigart M. A. Athanasiou K. A. (2005). Biomechanical characteristics of the normal medial and lateral porcine knee menisci. Proc. Inst. Mech. Eng. Part H J. Eng. Med. 219, 53–62. 10.1243/095441105X9174 15777057

[B26] Sweigart M. A. Zhu C. F. Burt D. M. DeHoll P. D. Agrawal C. M. Clanton T. O.. (2004). Intraspecies and interspecies comparison of the compressive properties of the medial meniscus. Ann. Biomed. Eng. 32, 1569–1579. 10.1114/B:ABME.0000049040.70767.5c 15636116

[B27] Travascio F. Devaux F. Volz M. Jackson A. R. (2020). Molecular and macromolecular diffusion in human meniscus: relationships with tissue structure and composition. Osteoarthritis Cartilage 28, 375–382. 10.1016/j.joca.2019.12.006 31917232 PMC7248550

[B28] Tsujii A. Nakamura N. Horibe S. (2017). Age-related changes in the knee meniscus. Knee 24, 1262–1270. 10.1016/j.knee.2017.08.001 28970119

[B29] Warnecke D. Balko J. Haas J. Bieger R. Leucht F. Wolf N.. (2020). Degeneration alters the biomechanical properties and structural composition of lateral human menisci. Osteoarthritis Cartilage 28, 1482–1491. 10.1016/j.joca.2020.07.004 32739340

[B30] Williamson A. K. Chen A. C. Sah R. L. (2001). Compressive properties and function—composition relationships of developing bovine articular cartilage. J. Orthop. Res. 19, 1113–1121. 10.1016/S0736-0266(01)00052-3 11781013

[B31] Yang N. H. Canavan P. K. Nayeb-Hashemi H. Najafi B. Vaziri A. (2010). Protocol for constructing subject-specific biomechanical models of knee joint. Comput. Methods Biomech. Biomed. Eng. 13, 589–603. 10.1080/10255840903389989 20521186

